# SCF^β-TRCP^-mediated degradation of NEDD4 inhibits tumorigenesis through modulating the PTEN/Akt signaling pathway

**DOI:** 10.18632/oncotarget.1675

**Published:** 2014-01-20

**Authors:** Jia Liu, Lixin Wan, Pengda Liu, Hiroyuki Inuzuka, Jiankang Liu, Zhiwei Wang, Wenyi Wei

**Affiliations:** ^1^ Center for Mitochondrial Biology and Medicine, The Key Laboratory of Biomedical Information Engineering of Ministry of Education, School of Life Science and Technology and Frontier Institute of Life Science, FIST, Xi'an Jiaotong University, Xi'an, China; ^2^ Department of Pathology, Beth Israel Deaconess Medical Center, Harvard Medical School, Boston, MA; ^3^ The Cyrus Tang Hematology Center, Jiangsu Institute of Hematology, the First Affiliated Hospital, Soochow University, Suzhou, Jiangsu, P. R. China

**Keywords:** β-TRCP, NEDD4, degradation, PTEN, Akt, cancer, phosphorylation, ubiquitination, therapy

## Abstract

The HECT domain-containing ubiquitin E3 ligase NEDD4 is widely expressed in mammalian tissues and plays a crucial role in governing a wide spectrum of cellular processes including cell growth, tissue development and homeostasis. Recent reports have indicated that NEDD4 might facilitate tumorigenesis through targeted degradation of multiple tumor suppressor proteins including PTEN. However, the molecular mechanism by which NEDD4 stability is regulated has not been fully elucidated. Here we report that SCF^β-TRCP^ governs NEDD4 protein stability by targeting it for ubiquitination and subsequent degradation in a Casein Kinase-I (CKI) phosphorylation-dependent manner. Specifically, depletion of β-TRCP, or inactivation of CKI, stabilized NEDD4, leading to down-regulation of its ubiquitin target PTEN and subsequent activation of the mTOR/Akt oncogenic pathway. Furthermore, we found that CKIδ-mediated phosphorylation of Ser347 and Ser348 on NEDD4 promoted its interaction with SCF^β-TRCP^ for subsequent ubiquitination and degradation. As a result, compared to ectopic expression of wild-type NEDD4, introducing a non-degradable NEDD4 (S347A/S348A-NEDD4) promoted cancer cell growth and migration. Hence, our findings revealed the CKI/SCF^β-TRCP^ signaling axis as the upstream negative regulator of NEDD4, and further suggested that enhancing NEDD4 degradation, presumably with CKI or SCF^β-TRCP^ agonists, could be a promising strategy for treating human cancers.

## INTRODUCTION

Ubiquitination by the UPS (Ubiquitin Proteasome System) is a post-translational modification that has been demonstrated to regulate various cellular processes including cell proliferation, cell cycle progression and migration [1]. It is well known that the UPS consists of the E1 ubiquitin-activating enzyme, the E2 ubiquitin-conjugating enzyme and the E3 ubiquitin ligase that interacts directly with the ubiquitin substrates [1]. As such, the specificity of targeted substrates is mainly determined by the E3 ligases [2]. E3 ubiquitin ligases are divided into multiple classes according to their functional domains, such as the Really Interesting New Gene (RING) type, Homologous to E6-AP Carboxyl-Terminus (HECT) type and Ring/Cullin Ligase (RCL)-type of E3 ligases. The E1 enzyme activates the ubiquitin molecule and transfers it to the E2 enzyme. Subsequently, the charged E2 enzyme binds a specific E3 ligase and transfers the ubiquitin directly to the substrates recruited by the E3 ligase. However, for HECT-type E3 ligases including NEDD4, the ubiquitin molecule is transiently transferred from the charged E2 enzyme to a specific HECT domain-containing E3 ligase, which then relays the ubiquitin molecule to its substrates [1]. Ubiquitin-conjugated substrates have distinct fates depending on the linkage of their conjugated ubiquitin or poly-ubiquitin chain [3]. Specifically, Lys48-linkage or Lys11-linkage poly-ubiquitin chains are recognized by the 26S proteasome for degradation [4]. On the other hand, Lys63-linkage or linear (M1)-linkage chains were recently found to be functionally linked to enzymatic activation or DNA damage repair processes [5], whereas the functional significance of other atypical ubiquitin linkages, including Lys6-linkage, Lys27-linkage, Lys29-linkage and Lys33-linkage, remain largely undefined [5].

The neural precursor cell-expressed developmentally downregulated gene 4 (NEDD4, also known as NEDD4-1) is the founding member of the family of HECT-type E3 ligases [6]. The NEDD4 protein contains three functional domains: a C-terminal HECT domain for E2 binding and ubiquitin loading, an N-terminal C2 domain for membrane attachment, and a central WW domain largely mediating its interaction with substrates [7]. NEDD4 was initially identified to play a critical role in regulating neuronal function and plasticity in the brain [8]. It is noteworthy that NEDD4 also exerts its functions in protein trafficking by recycling proteins through the endocytic machinery [6]. Biochemically, multiple substrates of NEDD4 have been discovered including ENaC (epithelial sodium channel) [9], AMPA (Amino-3-hydroxy-5-methyl-isoxazole-4-propionic acid) receptor [10, 11], Notch [12], IGF-1R (insulin-like growth factor-1 receptor) [13], VEGF-R2 (vascular endothelial growth factor receptor-2) [14], Cbl-b [15], Deltex [12], EPS15 (epithelial growth factor receptor substrate 15) [16], Spy1A [17] and PTEN (phosphatase and tensin homologue) [18, 19]. The identification of these downstream targets have helped further understanding the physiological functions of NEDD4 in various cellular processes. For example, NEDD4 was found to govern sodium homeostasis largely through negatively controlling the expression levels of ENaC [9]. Moreover, it has been reported that NEDD4 antagonizes Notch signaling by promoting the degradation of Notch and Deltex [12]. Notably, multiple laboratories recently identified a possible oncogenic role for NEDD4 in part by promoting the ubiquitination of the PTEN tumor suppressor [18, 19] to govern either its stability [19] or its subcellular localization [18]. Therefore, the NEDD4 E3 ligase might exert its physiological function largely through promoting the degradation of a broad range of downstream substrates.

Given its critical role in many key cellular processes, recent reports have suggested that NEDD4 could be regulated by multiple means [20]. For instance, NEDD4 was found to be phosphorylated by Src [16], and could be cleaved by several caspases during apoptosis [21]. Furthermore, consistent with a possible oncogenic role for NEDD4, overexpression of NEDD4 was observed in human cancers such as prostate, bladder [22], colorectal [12] and non-small-cell lung carcinomas (NSCLC) [23] and promoted growth of colon cancer cells [12]. Therefore, targeted reduction of NEDD4 expression might be a promising strategy for treatment of these human cancers. However, it remains largely unknown how NEDD4 stability is regulated physiologically, and how it becomes aberrantly upregulated in human cancers. In this study, we explored the molecular mechanisms underlying NEDD4 stability control and further examined whether alterations in regulation of NEDD4 expression contributes to tumorigenesis. To this end, our results identify that the ubiquitin E3 ligase SCF^β-TRCP^ governs NEDD4 protein stability by targeting NEDD4 for ubiquitination and subsequent destruction in a CKI-dependent manner. More importantly, dysregulated NEDD4 degradation led to reduction in PTEN expression and subsequent hyper-activation of the oncogenic mTOR/Akt pathway, resulting in enhanced tumor cell proliferation and growth.

## RESULTS

### NEDD4 binds the SCF^β-TRCP^ ubiquitin E3 ligase complex

**Figure 1:**
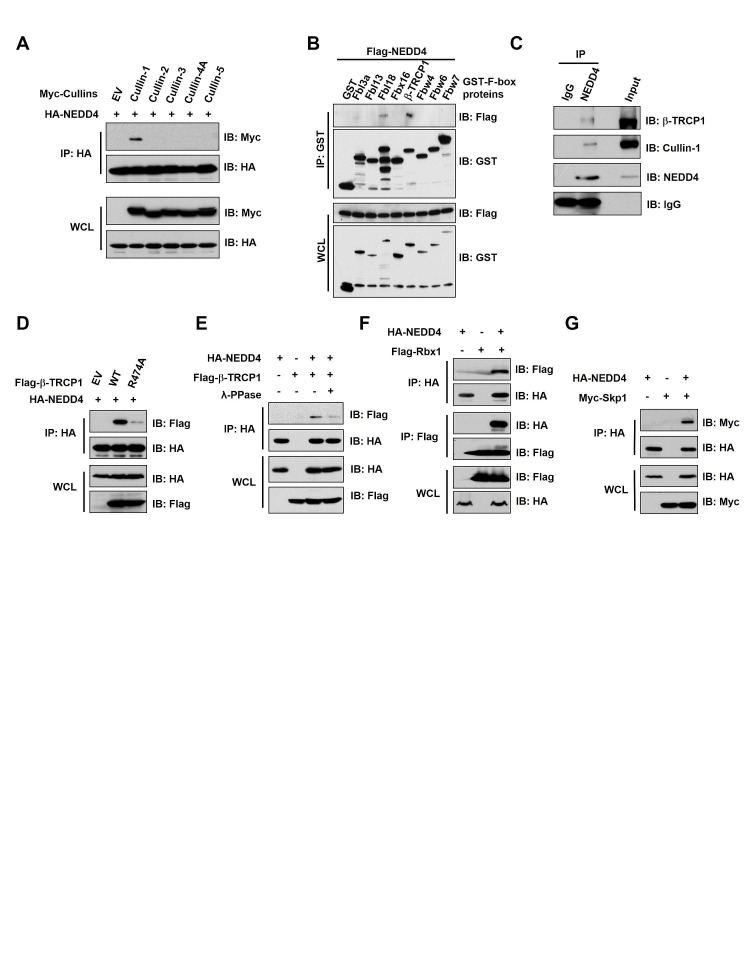
NEDD4 interacted with the SCF^β-TRCP^ ubiquitin E3 ligase complex (A) Immunoblot (IB) analysis of whole cell lysates (WCL) and immunoprecipitates (IP) derived from 293T cells transfected with Myc-tagged Cullin constructs or empty vector (EV) as a negative control. (B) IB analysis of WCL and IP derived from 293T cells transfected with Flag-NEDD4 and the indicated GST-F-box protein constructs. (C) HeLa cell extracts were immunoprecipitated with antibody against NEDD4, or control IgG and analyzed by IB analysis. (D) IB analysis of WCL and IP derived from 293T cells transfected with HA-NEDD4 and Flag-tagged wild-type or R474A mutant β-TRCP1 constructs, or EV as a negative control. (E) IB analysis of WCL and IP derived from 293T cells transfected with HA-NEDD4 and Flag-β-TRCP1 constructs. Where indicated, cell lysates were pre-treated with λ-phosphatase before the IP procedure. (F) IB analysis of WCL and IP derived from 293T cells transfected with HA-NEDD4 and Flag-Rbx1 constructs as indicated. (G) IB analysis of WCL and IP derived from 239T cells transfected with HA-NEDD4 and Myc-Skp1 constructs as indicated.

It is well known that Cullin-Ring complexes comprise the largest known group of E3 ubiquitin ligases [24]. Therefore, we first explored whether a specific Cullin-Ring E3 ligase might be involved in the regulation of NEDD4 destruction. By examining a panel of Cullin members, we observed that NEDD4 interacted with Cullin-1, but not Cullin-2, Cullin-3, Cullin-4A or Cullin-5 (Figure 1A). Furthermore, among the F-box proteins examined, NEDD4 bound β-TRCP1 (Figure 1B), and to a much lesser extent, Fbl18 (Figure 1B). Consistent with this notion, NEDD4 interacted with β-TRCP1 at both endogenous (Figure 1C) and exogenous levels (Figure 1D). More importantly, a mutant form of β-TRCP1 (R474A) deficient in binding substrates [25] exhibited significantly reduced ability to bind NEDD4 (Figure 1D and Supplementary Figure 1), supporting a specific and direct interaction between β-TRCP1 and NEDD4. Consistent with a role for substrate phosphorylation for β-TRCP recognition [26-29], phosphatase treatment led to a reduction in NEDD4 interaction with β-TRCP1 (Figure 1E). Notably, we also observed that NEDD4 interacted with the SCF E3 ligase components Rbx1 (Figure 1F) and Skp1 (Figure 1G), further indicating the possible involvement of the complete SCF^β-TRCP^ complex in regulating NEDD4 abundance.

### Depletion of β-TRCP led to increased NEDD4 protein levels

**Figure 2:**
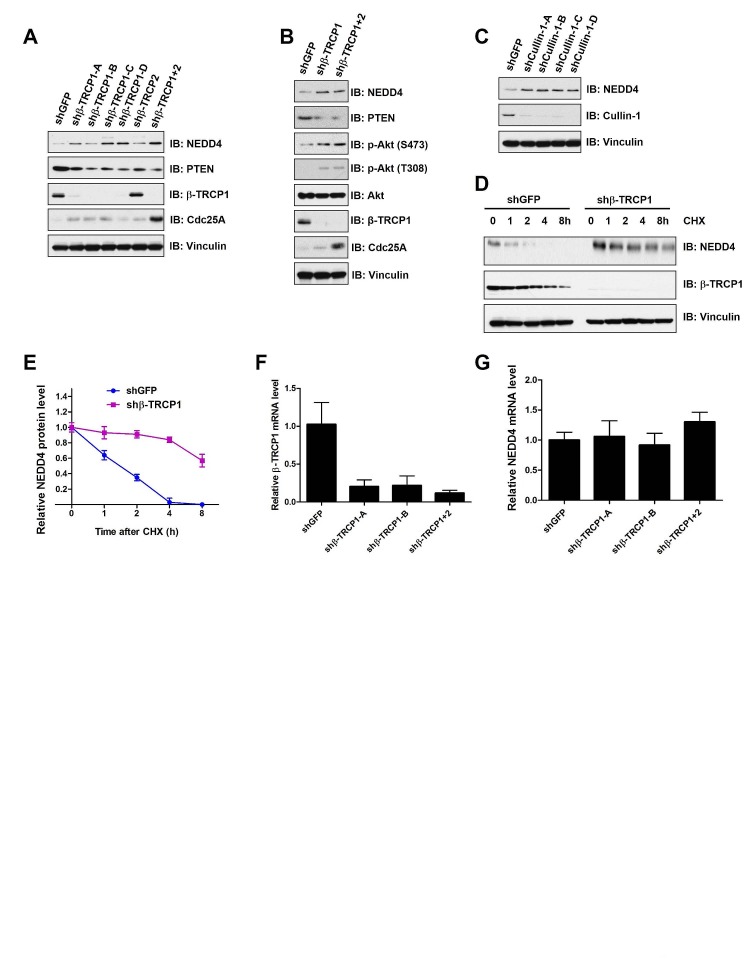
Depletion of β-TRCP increased NEDD4 protein levels (A) Immunoblot (IB) analysis of whole cell lysates (WCL) derived from HeLa cells infected with shRNA constructs specific for GFP, β-TRCP1 (four independent lentiviral β-TRCP1-targeting shRNA constructs named -A, -B, -C, -D), β-TRCP2 or β-TRCP1+2, followed by selection with 1 μg/ml puromycin for three days to eliminate the non-infected cells. (B) IB analysis of WCL derived from U2OS cells infected with shRNA constructs specific for GFP, β-TRCP1 or β-TRCP1+2, followed by selection with 1 μg/ml puromycin for three days to eliminate non-infected cells. (C) IB analysis of WCL from 293T cells transfected with shRNA specific for GFP or several shRNA constructs against Cullin 1 (four independent lentiviral Cullin 1-targeting shRNA constructs named -A, -B, -C, -D) followed by selection with 1 μg/ml puromycin for three days to eliminate non-infected cells. (D) HeLa cells were infected with the shRNA constructs for GFP or β-TRCP1 followed by selection with 1 μg/ml puromycin for three days to eliminate non-infected cells. The generated stable cell lines were then split into 60-mm dishes. 20 hours later, cells were treated with 20μg/ml cycloheximide (CHX). At the indicated time points, WCLs were prepared and immunoblots were probed with the indicated antibodies. (E) Quantification of the band intensities in (D). NEDD4 band intensity was normalized to Vinculin, and then normalized to the t = 0 controls. The error bars represent mean ± SD (*n* = 3). (F-G) Relative mRNA levels of β-TRCP1 (F) or NEDD4 (G) in HeLa cells infected with shRNA constructs specific for GFP, β-TRCP1 (-A and -B) or β-TRCP1+2 followed by selection with 1 μg/ml puromycin for three days to eliminate the non-infected cells. NEDD4 and β-TRCP1 mRNA levels were normalized to GAPDH, and then normalized to the control cells (shGFP). The error bars represent mean ± SD (*n* = 3).

To validate whether NEDD4 is a potential physiological substrate for β-TRCP, we examined NEDD4 protein abundance in various cell lines after depleting endogenous β-TRCP by multiple independent shRNAs (Supplementary Figures 2A-B). We observed that depletion of endogenous β-TRCP1, or both β-TRCP1 and β-TRCP2 (β-TRCP1+2)), led to the upregulation of NEDD4 protein levels in HeLa (Figure 2A), U2OS (Figure 2B) and 293T cells (Supplementary Figure 2C). Furthermore, in keeping with previous reports that NEDD4 is a proto-oncogenic ubiquitin ligase for the tumor suppressor PTEN [18, 19], we observed that depletion of β-TRCP led to decreased levels of PTEN (Figures 2A and 2B) and subsequent elevated phosphorylation of Akt (Figure 2B). In further support of NEDD4 as a putative ubiquitin substrate for SCF^β-TRCP^, depletion of endogenous Cullin1 also led to an accumulation of NEDD4 (Figure 2C and Supplementary Figure 2D). Moreover, consistent with a negative role of β-TRCP in regulating NEDD4 stability, depletion of endogenous β-TRCP1 dramatically increased NEDD4 protein half-life (Figures 2D and 2E) without significantly affecting NEDD4 mRNA levels (Figures 2F and 2G). Taken together, these results indicated that SCF^β-TRCP^ negatively regulates NEDD4 stability.

### Casein Kinase Iδ (CKIδ) promoted the destruction of NEDD4

**Figure 3:**
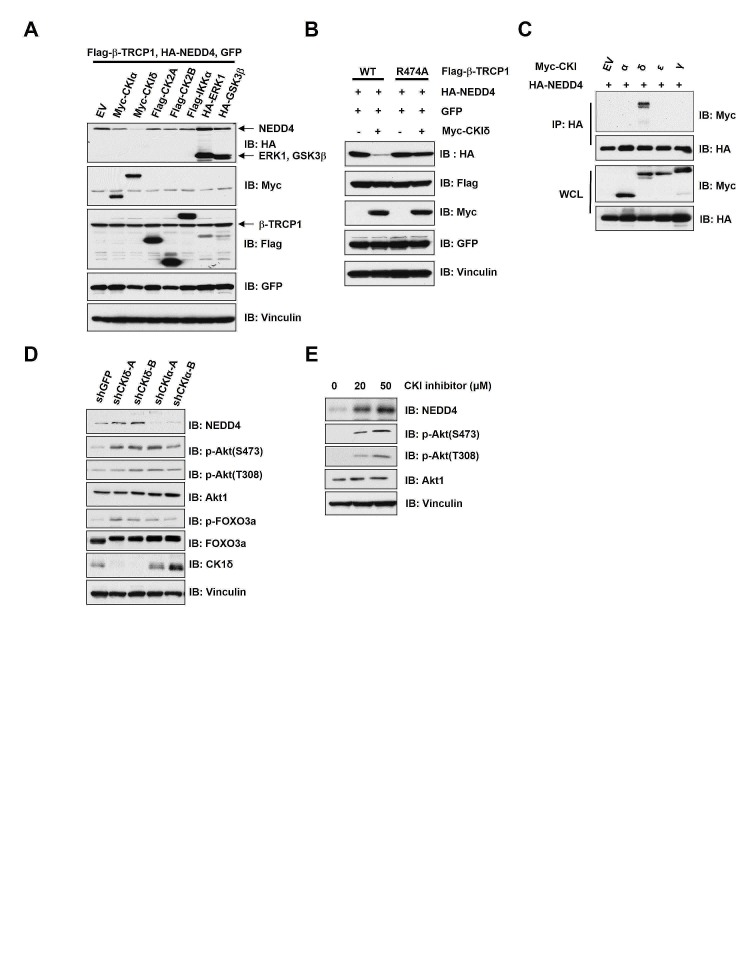
Casein Kinase Iδ (CKIδ) negatively controlled NEDD4 stability (A) Immunoblot (IB) analysis of whole cell lysates (WCL) derived from 293T cells transfected with HA-NEDD4, Flag-β-TRCP1 and the indicated kinases. (B) IB analysis of WCL derived from 239T cells transfected with HA-NEDD4 and/or Myc-CKIδ together with Flag-WT–β-TRCP1 or Flag-R474A–β-TRCP1. (C) IB analysis of WCL and immunoprecipitates (IP) derived from HeLa cells transfected with HA-NEDD4 and Myc-tagged versions of the indicated CKI isoforms. (D) IB analysis of HeLa cells that were infected with shRNA specific for GFP or the indicated CKI isoforms, followed by selection with 1 μg/ml puromycin for three days to eliminate non-infected cells. (E) IB analysis of 293T cells treated with the CKI inhibitor D4476 at the indicated concentrations for 12 hours.

As proper phosphorylation of substrates by specific kinase(s) is required for their recognition by the SCF^β-TRCP^ E3 ligase for subsequent ubiquitination and destruction [1], we explored the candidate kinases that are possibly involved in regulating the degradation of NEDD4 by SCF^β-TRCP^. To this end, we found that ectopic expression of CKIδ, but not CKIα, CK2, IKKα, ERK or GSK3β significantly reduced NEDD4 protein abundance in β-TRCP1-WT (Figure 3A), but not the β-TRCP1-R474A mutant (Figure 3B) expressing cells. These results demonstrated that CKIδ might be the specific kinase that phosphorylates NEDD4 to trigger its interaction with β-TRCP. Furthermore, we observed that only CKIδ, but not CKIα, CKIε or CKIγ interacted with NEDD4, further pinpointing CKIδ as the specific CKI isoform that might function as the upstream modifying enzyme to trigger ubiquitination of NEDD4 by SCF^β-TRCP^ (Figure 3C). To further validate the critical role of CKIδ in controlling NEDD4 stability, we inactivated CKIδ in HeLa cells by either shRNA-mediated depletion of endogenous CKI (Figure 3D) or by a CKI specific pharmacological inhibitor, D4476 (Figure 3E), both of which led to elevated expression of NEDD4 (Figures 3D and 3E). These results cumulatively demonstrated that CKIδ might play a pivotal role in triggering the destruction of NEDD4 by SCF^β-TRCP^.

### CKIδ phosphorylated NEDD4 at both S347 and S348 to promote the ubiquitination and destruction of NEDD4 by SCF^β-TRCP^

**Figure 4:**
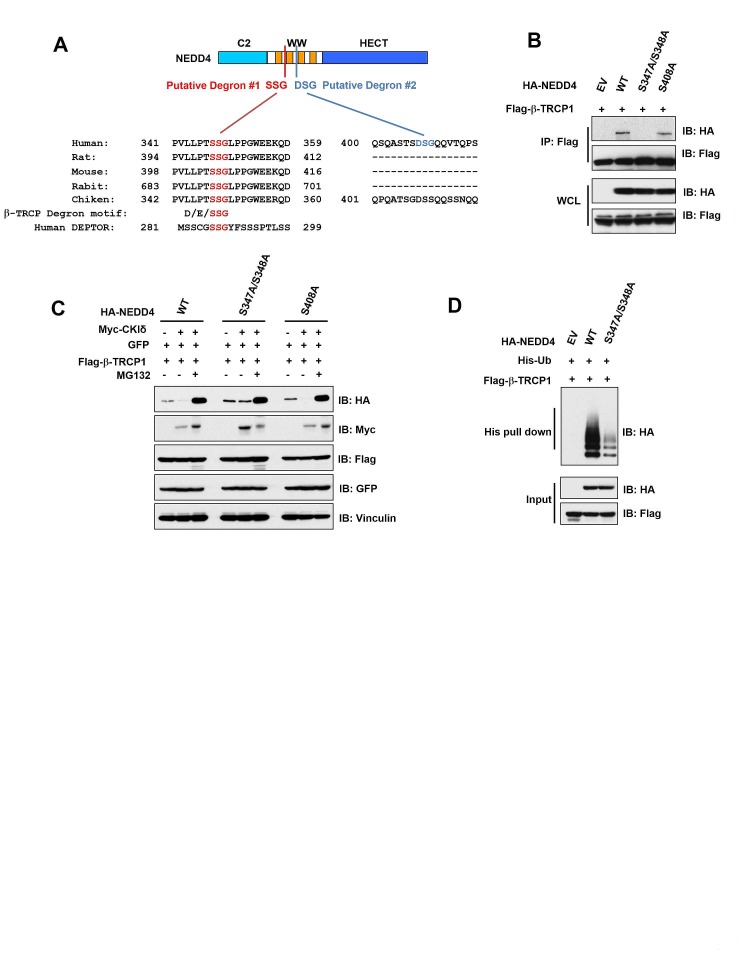
CKIδ phosphorylated NEDD4 at S347/S348 sites to facilitate its ubiquitination and subsequent destruction by SCF^β-TRCP^ (A) Alignment of the candidate phospho-degron sequences in NEDD4 from different species. (B) Immunoblot (IB) analysis of whole cell lysates (WCL) and immunoprecipitates (IP) derived from HeLa cells transfected with Flag–β-TRCP1 together with HA-WT-NEDD4, HA-S408A-NEDD4 or HA-S347A/S348A-NEDD4. (C) IB analysis of HeLa cells transfected with Flag-β-TRCP1 and HA-tagged wild-type, S408A, or S347A/S348A mutant forms of NEDD4. Where indicated, cells were treated with Myc-CKIδ, or treated with the proteasome inhibitor MG132 before harvesting. D) IB analysis of WCL and IP derived from HeLa cells transfected with Flag-β-TRCP1, His-Ubiquitin, and HA-tagged wild-type or S347A/S348A mutant NEDD4 constructs, or EV (as a negative control) as indicated.

It has been demonstrated that most β-TRCP substrates contain a canonical DSGxxS degron sequence [30]. Unexpectedly, although human NEDD4 contains a canonical DSGxxS degron (Degron #2), it is not conserved among various species including rat, mouse, rabbit and chicken (Figure 4A). However, NEDD4 contains an evolutionally degenerate phospho-degron variant SSG (termed Degron #1, Figure 4A and Supplementary Figure 3A), which is also present in DEPTOR, a well characterized β-TRCP substrate [27, 31, 32]. Notably, Degron #1 exists in all four isoforms of human NEDD4 (Supplementary Figure 3A), Given that isoform 4 of NEDD4 for has been previously described as a functional isoform [33], we used this isoform for all our studies. To determine which degron, when phosphorylated, is critical for governing NEDD4 stability, we mutated these Serine-347/Serine-348 (within Degron #1), Serine-408 (within Degron #2) to alanines to generate phospho-deficient mutants. Interestingly, we found that the S347A/S348A, but not the S408A mutation, led to a significantly decreased interaction between NEDD4 and β-TRCP1 (Figure 4B and Supplementary Figure 3B), arguing for a critical role for the phosphorylation of S347/S348 in β-TRCP-mediated destruction of NEDD4. Consistently, compared to NEDD4-WT, the NEDD4-S347A/S348A mutant displayed resistance to β-TRCP1/CKIδ-mediated degradation (Figure 4C). Moreover, this degradation process was efficiently blocked by the proteasome inhibitor MG132, indicating the involvement of the 26S proteasome and ubiquitin system in β-TRCP1/CKIδ-mediated degradation of NEDD4 (Figure 4C). More importantly, compared to NEDD4-WT, the NEDD4-S347A/S348A mutant was deficient in undergoing poly-ubiquitination as detected by *in vivo* ubiquitination assays (Figure 4D). Taken together, our results indicate that CKIδ can phosphorylate NEDD4 at the S347/S348 sites within the putative degenerate Degron #1, to trigger the interaction of NEDD4 with β-TRCP for subsequent ubiquitination and destruction events.

### Loss of β-TRCP-mediated degradation of NEDD4 promoted cancer cell growth and migration

**Figure 5:**
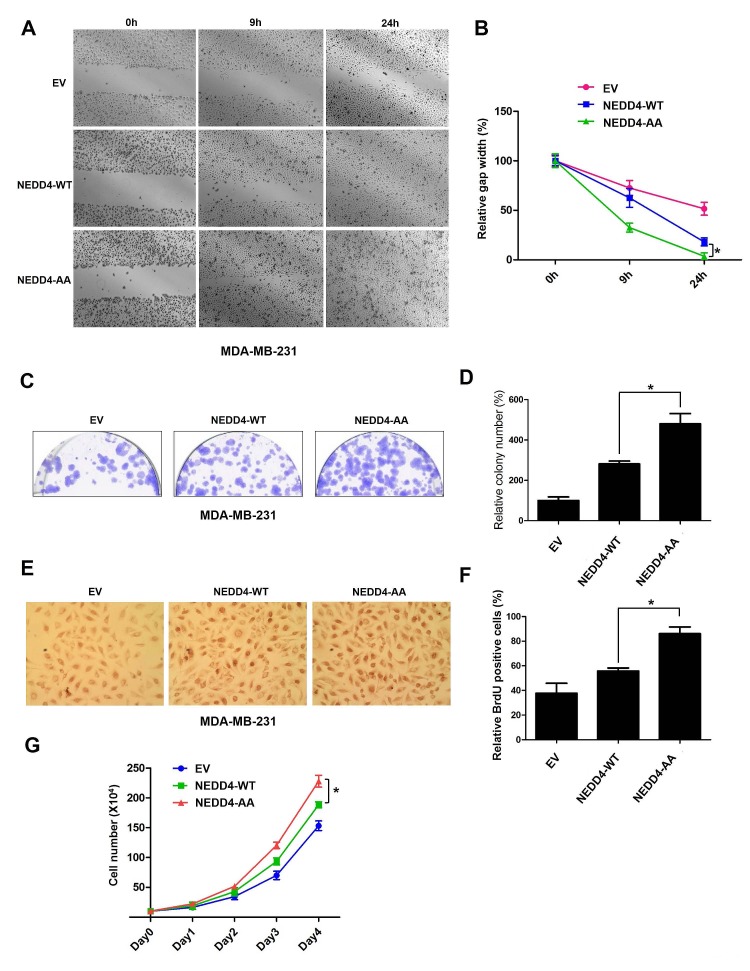
Loss of SCF^β-TRCP^-mediated degradation of NEDD4 promoted cancer cell proliferation and migration (A-B) Scratch assays were performed with MDA-MB-231 cells that were infected with EV, HA-wild-type (WT)-NEDD4 or HA-S347A/S348A (AA)-NEDD4 encoding retroviral vectors followed by 3 days of puromycin (1 μg/ml) selection to eliminate non-infected cells. The generated various MDA-MB-231 cell lines were seeded on a 6-well plate and scratched on the surface with a pipette tip. Relative values were set at 1 for the gap width at the time of the scratch. Representative photographs at time points 0, 9 and 24 hours after the scratch (A). Measurements were done in duplicate in 3 separate experiments, and data were depicted as average gap width (B). The error bars represented mean ± SD (*n* = 3), * *p*<0.05 (Student's *t*-test), compared with cells expressing WT-NEDD4. (C-D) Colony formation assays were performed with the MDA-MB-231 cell lines generated in (A). After 4 days, the colonies were stained with crystal violet and counted (C). The number of surviving colonies were calculated as the average of triplicates (D). The error bars represented mean ± SD (*n* = 3), * *p*<0.05 (Student's *t*-test), compared with cells expressing WT-NEDD4. (E-F) BrdU labeling analysis was performed with MDA-MB-231 cell lines generated in (A). Cells were incubated with BrdU and uridine for 48 hours and representative photographs were taken (E). Percentage of BrdU positive cells was illustrated in (F). The error bars represented mean ± SD (*n* = 3), * *p*<0.05 (Student's *t*-test), compared with cells expressing WT-NEDD4. (G) MDA-MB-231 cells stably expressing control EV, HA-WT-NEDD4 or HA-S347A/S348A-NEDD4 were seeded and analyzed for cell proliferation capacities. The data shown are derived from three independent experiments, and the error bars represented mean ± SD. (* *p*<0.05, compared with cells expressing WT-NEDD4; Student's *t*-test).

**Figure 6:**
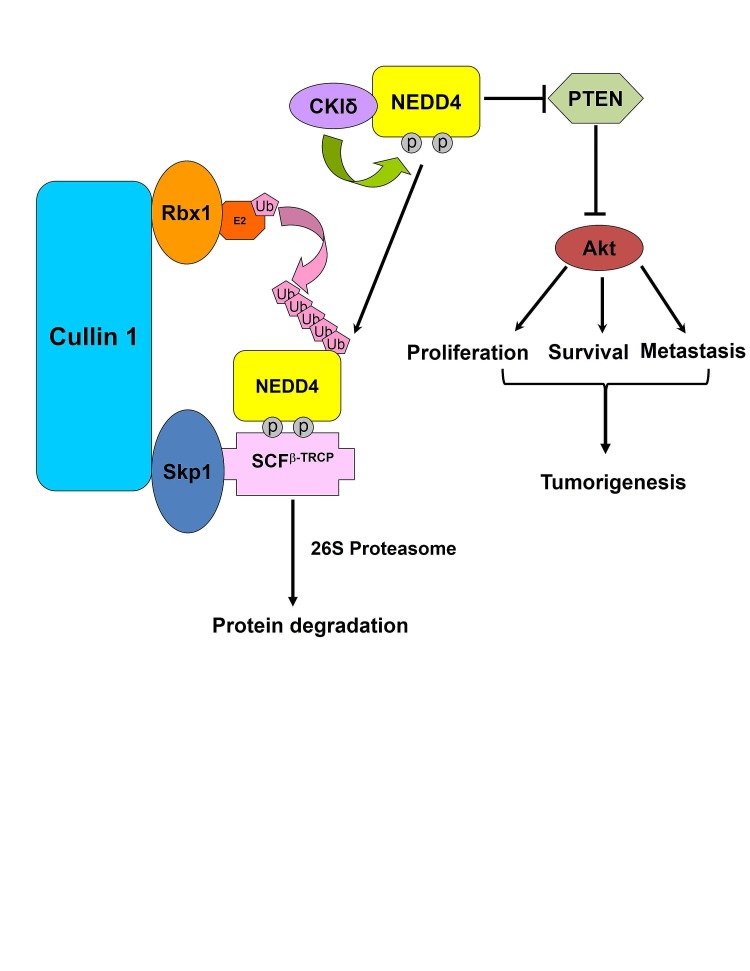
A proposed model for how bypassing SCF^β-TRCP^-mediated ubiquitination and degradation of the NEDD4 oncoprotein might promote tumorigenesis in part through elevating the Akt oncogenic signaling pathway by downregulating the PTEN tumor suppressor

It has been reported that NEDD4 plays an important role in facilitating the progression of breast and prostate cancers [22, 34]. Therefore, we next explored whether β-TRCP1-mediated NEDD4 destruction is involved in cancer cell growth and migration. To this end, we retro-virally expressed a non-degradable mutant form of NEDD4 (NEDD4-S347A/S348A, referred to as NEDD4-AA) or wild-type NEDD4 (NEDD4-WT) in the breast cancer cell line MDA-MB-231 and the prostate cancer cell line DU145. Notably, we found that NEDD4-AA expressing MDA-MB-231 cells and DU145 cells displayed an enhanced migration ability compared to NEDD4-WT or EV infected cells (Figures 5A and 5B). Furthermore, NEDD4-AA expressing cells formed more colonies in soft agar (Figures 5C, 5D and Supplementary Figures 4A-B) and displayed elevated S phase entry (Figures 5E, 5F and Supplementary Figures 4C-D) than cells infected with NEDD4-WT or EV controls. Consistent with the observation that depletion of β-TRCP led to PTEN down-regulation and subsequent elevation of p-Akt largely via stabilizing NEDD4 (Figure 2B), we found that in both MDA-MB-231 and DU145 cells, ectopic expression of the non-degradable NEDD4 mutant (NEDD4-AA) reduced PTEN expression that subsequently led to Akt activation (Supplementary Figure 4E-F). These results provided a possible mechanism for the observed enhanced tumorigenicity of NEDD4-AA-expressing MDA-MB-231 and DU145 cells (Figures 5A-G, and Supplementary Figures 4A-D). Taken together, these results suggest that by evading β-TRCP-mediated degradation, cells expressing the non-degradable version of NEDD4 (NEDD4-AA) have increased cell growth and cell migration, presumably through the activated mTOR/Akt pathway by suppressing the PTEN tumor suppressor. Furthermore, as illustrated in Figure 5G, ectopic expression of NEDD4-AA in MDA-MB-231 cells also significantly enhanced cell proliferation. Altogether, these data suggest that β-TRCP-mediated destruction of the NEDD4 oncoprotein could inhibit cell proliferation and migration and bypassing this negative regulation could possibly facilitate tumorigenesis (Figure 6).

## DISCUSSION

In the present study, for the first time, we provide evidence for a mechanism by which SCF^β-TRCP^ controls NEDD4 protein stability in a CKIδ-dependent manner. In support of this concept, we observed that depletion of β-TRCP (Figures 2A-B) or inactivation of CKIδ (Figures 3A and 3D-E) led to accumulation of NEDD4 and subsequent down-regulation of its well characterized substrate, PTEN, leading to the activation of the downstream oncogenic mTOR/Akt pathway. More importantly, our results showed that CKIδ phosphorylates NEDD4 at both the Ser347 and Ser348 sites to trigger NEDD4 ubiquitination by SCF^β-TRCP^ and subsequent destruction by the 26S proteasome (Figures 4B and 4C). Consistent with a critical role of NEDD4 phosphorylation in triggering its degradation, introducing a non-degradable NEDD4 mutant (S347A/S348A) promoted cancer cell growth and migration in both *PTEN* positive prostate and breast cancer cells (Figure 5). Our findings therefore demonstrate that NEDD4 may be a potential target in the treatment of human cancers.

Previous studies have shown that NEDD4 could be regulated by various means [35, 36]. For example, Forkhead box protein M1B (FoxM1B) promoted NEDD4 expression, leading to cellular transformation and full malignant phenotype in immortalized human astrocytes [35]. Similarly, NDRG1 (N-myc downstream regulated gene-1) could also regulate NEDD4 expression in pancreatic cancer cells [36]. Our study here demonstrated that besides transcriptional regulation, NEDD4 turnover is also controlled by β-TRCP in a CKI phosphorylation-dependent manner. Importantly, recent studies have begun to reveal that NEDD4 might function as an oncoprotein that is frequently overexpressed in human tumors [12, 22, 23]. Mechanistically, NEDD4 might exert its oncogenic role in part through degradation of its target proteins such as LATS1 and PTEN [18, 37]. Salah *et al.* reported that NEDD4 directly interacts with LATS1, leading to ubiquitination and decreased levels of LATS1, thus inhibiting the activity of the Hippo pathway [37]. Similarly, several studies have shown that NEDD4 is a PTEN negative regulator in human malignancies [23, 38-41]. In support of this notion, NEDD4 overexpression was observed to positively correlate with the loss of PTEN in human cancer [38, 41, 42]. In line with this finding, we found that depletion of β-TRCP caused increased NEDD4 abundance and a subsequent decrease in PTEN expression, which in turn led to hyper-activation of its downstream signaling component, Akt, to promote cell survival and proliferation.

NEDD4 has been previously found to be overexpressed in colorectal cancers and promoted growth of colon cancer cells [12]. Similarly, suppression of NEDD4 expression significantly inhibited proliferation of NSCLC cells *in vitro* and tumor growth *in vivo*, whereas NEDD4 overexpression augmented the tumorigenicity of lung cancer cells with an intact *PTEN* gene [23]. Consistent with these reports, we observed that ectopic expression of non-degradable NEDD4-AA significantly promoted cell growth compared to cells expressing WT-NEDD4 (Figures 5C-G). Notably, we found that cells expressing NEDD4-AA had increased cellular migration ability (Figures 5A-B). As NEDD4 negatively regulates PTEN stability, NEDD4 could possibly promote cell growth and migration partly through activation of the mTOR/Akt pathway in prostate and breast cancers. However, further in-depth investigation is required to explore whether NEDD4 promotes cell invasion and metastasis in other human cancers, and its dependence on activation of the mTOR/Akt oncogenic signaling pathway.

In summary, our results indicate that β-TRCP governs NEDD4 stability in a CKIδ phosphorylation-dependent manner. Therefore, drugs that could induce NEDD4 degradation through upregulated expression or activating -TRCP1 or CKIδ, could be useful to treat human cancers with high expression of NEDD4. Hence, our findings suggest that targeting NEDD4 destruction could be a promising approach to achieve better treatment outcomes in human cancers.

## MATERIALS AND METHODS

### Cell culture

DU145 cells were cultured in RPMI 1640 medium (Life Technologies, CA) supplemented with 10% FBS, penicillin and streptomycin. HeLa, 293T, MCF-7 and MDA-MB-231 cells were cultured in DMEM medium (Life Technologies, CA) supplemented with 10% FBS, penicillin and streptomycin. The cells were maintained in a 5% CO_2_-humidified atmosphere at 37°C, as previously described [26, 28, 29].

### Plasmids

PCI-HA-NEDD4 was obtained from Addgene (27002) [33]. Various NEDD4 mutants were generated using the QuikChange XL Site-Directed Mutagenesis Kit (Stratagene) according to the manufacturer's instructions. Lentiviral short hairpin RNAs (shRNA lentiviral vectors) against GFP, β-TRCP1, β-TRCP1+2 and various CKI isoforms were described previously [43, 44]. Flag-β-TRCP1 and Flag-β-TRCP1-R474A constructs were described previously [30, 45]. Myc-Cullin 1, Myc-Cullin 2, Myc-Cullin 3, Myc-Cullin 4 and Myc-Cullin 5 constructs were obtained from James DeCaprio (Dana-Farber Cancer Institute, Boston, MA). Lentiviral shRNA constructs against GFP and various CKI isoforms were gifts from William Hahn (Dana-Farber Cancer Institute, Boston, MA). shRNA lentiviral vectors against Cullin 1 were gifts from J. Wade Harper (Harvard Medical School, Boston, MA).

### Antibodies and reagents

Anti-NEDD4 (4013), anti-PTEN (9188), anti-Akt1 (2938), anti-pAkt (S473) (9018), anti-pAkt (T308) (13038), anti-p-FOXO3a (T32) (9464), anti-FOXO3a (2497) and anti-Cdc25A (3652) antibodies were purchased from Cell Signaling Technology. Anti-c-Myc (9E10) and polyclonal anti-HA antibodies (SC-805) were purchased from Santa Cruz Biotechnology. Anti-Vinculin antibody (V-4505), polyclonal anti-Flag antibody (F-2425), monoclonal anti-Flag antibody (F-3165), anti-HA agarose beads (A-2095), peroxidase-conjugated anti-mouse secondary antibody (A-4416) and peroxidase-conjugated anti-rabbit secondary antibody (A-4914) were purchased from Sigma. Anti-GFP antibody (632380) was obtained from Invitrogen. Anti-Cullin 1 (4995) and anti–β-TRCP1 (4394) antibodies were purchased from Cell Signaling Technology. Monoclonal anti-HA antibody (MMS-101P) was purchased from Covance. Oligofectamine, Lipofectamine and Plus reagents were purchased from Life Technologies.

### Immunoblot and immunoprecipitation

Cells were lysed in EBC lysis buffer (50 mM Tris pH 8.0, 120 mM NaCl, 0.5% NP-40) supplemented with protease inhibitors (Roche) and phosphatase inhibitors (EMD Millipore). The protein concentrations were measured using the Bio-Rad protein assay reagent (Bio-Rad Laboratories, CA). The lysates were then resolved by SDS-PAGE (sodium dodecyl sulfate polyacrylamide gel electrophoresis) and immunoblotted with indicated antibodies as described previously [26, 28, 29]. For immunoprecipitation assays, 800 μg lysates were incubated with the appropriate antibody (1-2 μg) overnight at 4 °C followed by addition of Protein A sepharose beads for one hour. Immunocomplexes were resolved by SDS-PAGE and immunoblotted with indicated antibodies after washed with NETN buffer (20 mM Tris, pH 8.0, 100 mM NaCl, 1 mM EDTA and 0.5% NP-40).

### Protein degradation analysis

Cells were transfected with plasmids encoding PCI-HA-NEDD4, Flag-β-TRCP1 and GFP as a transfection control, in the presence or absence of Myc-CKIδ. After 40 hours, cells were lysed and immunoblot analysis was performed. For half-life studies, 20 μg/ml cycloheximide (CHX, Sigma C7698) was added to the medium 40 hours post-transfection, cells were then lysed at the indicated time points and immunoblot analysis was conducted to detect protein abundances.

### In vivo ubiquitination assays

Cells were transfected with His-Ubiquitin along with HA-NEDD4 (wild-type or S347A/S348A) and Flag-β-TRCP1. 36 hours post-transfection, cell lysates were incubated with Ni-NTA matrices (Qiagen) at 4 °C for 12 hours in the presence of 8 M Urea (pH 7.5) as described previously [46]. Then, immobilized proteins were immunoblotted with the anti-HA antibody after washed five times with 8 M urea at pH 6.3.

### Cell transfection

Cells were transfected using Lipofectamine (Life Technologies) in OptiMEM medium (Life Technologies) according to the manufacturer's instructions. 48 hours post-transfection, transfected cells were further subjected to Real-time RT-PCR or immunoblot analysis to detect the efficacy of transfection.

### Scratch assays

Cancer cells were cultured in 6-well plate until the cells grew to confluence. The scratch wound was scraped in a straight line using a pipette tip. Photographic images were taken at 0 hour and 18 hours. Gap width measurements were conducted with Photoshop CS4 using the analytical ruler tool.

### Colony formation assays

Cancer cells were seeded into gelatinized plates. After 7 days, the colonies were stained with crystal violet and counted. The numbers of surviving colonies were calculated as the average of triplicates as described previously [26].

### Bromodeoxyuridine (BrdU) labeling assays

Cells were incubated with BrdU and uridine for 48 hours. Then the BrdU labeling assay was performed as described previously [47].

### Real-time RT-PCR

RNA was extracted using the Qiagen RNeasy mini kit, and the reverse transcription reaction was performed using the ABI Taqman Reverse Transcription Reagent (N808-0234). The real-time RT-PCR reaction was performed with the ABI-7500 Fast Real-time PCR system as described previously [48].

### Statistical Analysis

All quantitative data were presented as the mean ± SD as indicated of at least three independent experiments by Student's *t* test for between group differences. *p* < 0.05 was considered as statistically significant.

## SUPPLEMENTARY FIGURES


