# Supplementary material for: SCFβ-TRCP-mediated degradation of NEDD4 inhibits tumorigenesis through modulating the PTEN/Akt signaling pathway
Source: Oncotarget. 2014 Jan 20;5(4):1026–37. doi: 10.18632/oncotarget.1675 (PMC4011580; doi:10.18632/oncotarget.1675)
Supplement: Supplementary file 1 [file oncotarget-05-1026-s001.doc]

**SCF^β-TRCP^-mediated degradation of NEDD4 inhibits tumorigenesis through modulating the PTEN/Akt signaling pathway**

Jia Liu^1,2,*^ , Lixin Wan^2,*^, Pengda Liu^2,*^, Hiroyuki Inuzuka^2^, Jiankang Liu^2^, Zhiwei Wang^3,#^

and Wenyi Wei^2,#^

*^1^ Center for Mitochondrial Biology and Medicine, The Key Laboratory of Biomedical Information Engineering of Ministry of Education, School of Life Science and Technology and Frontier Institute of Life Science, FIST, Xi’an Jiaotong University, Xi’an 710049, China*

*^2^ Department of Pathology, Beth Israel Deaconess Medical Center, Harvard Medical School,*

*Boston, MA 02215*

*^3^ The Cyrus Tang Hematology Center, Jiangsu Institute of Hematology, the First Affiliated Hospital, Soochow University, Suzhou, Jiangsu 215123, P. R. China*

**These three authors contributed equally to this work*

**Supplementary Figures S1-S4**


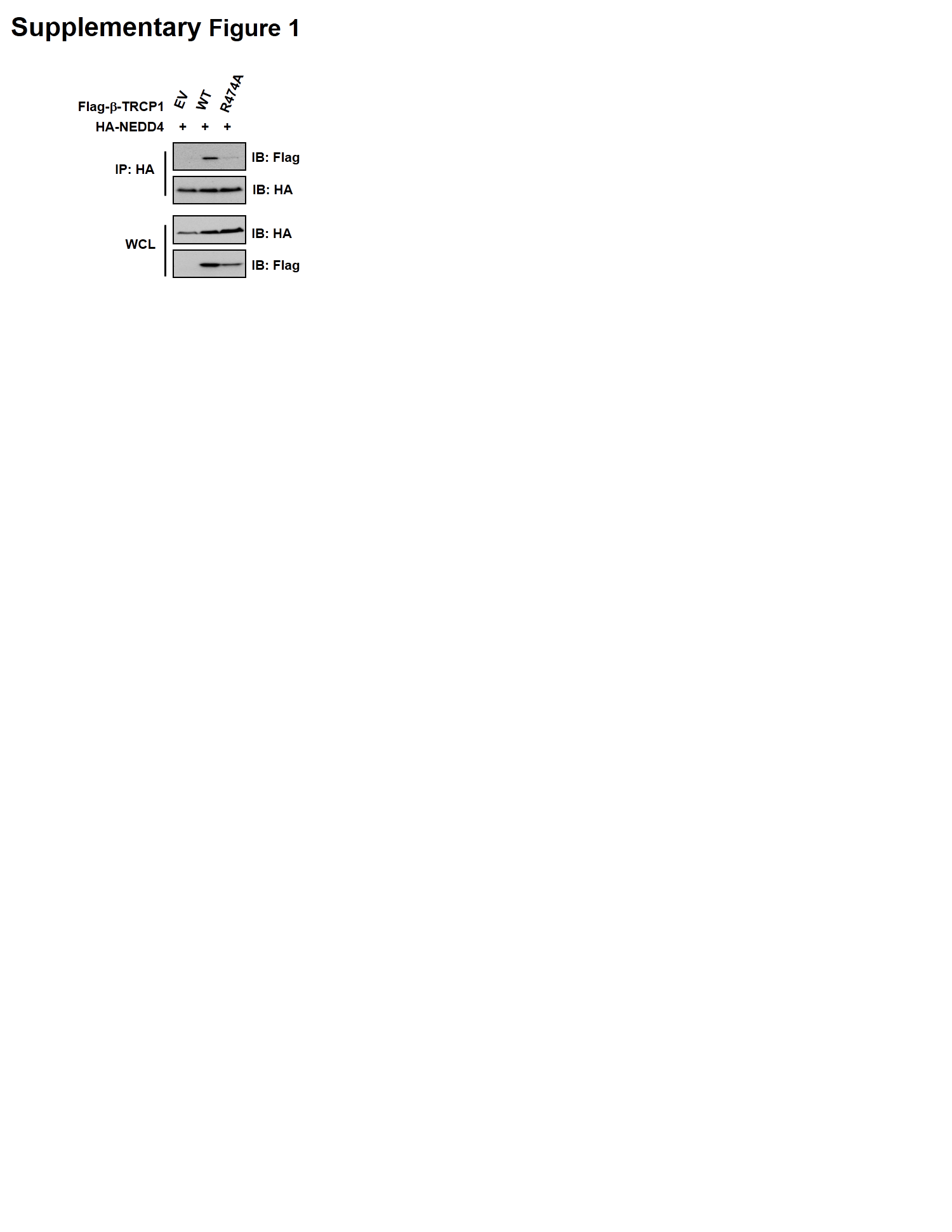


**Supplementary Figure 1**. **NEDD4 interacted with β-TRCP1**.

Immunoblot analysis (IB) of whole cell lysates (WCL) and immunoprecipitates (IP) derived from HeLa cells transfected with HA-NEDD4 and Flag-tagged wild-type (WT) or R474A mutant β-TRCP1 constructs, or EV as a negative control.


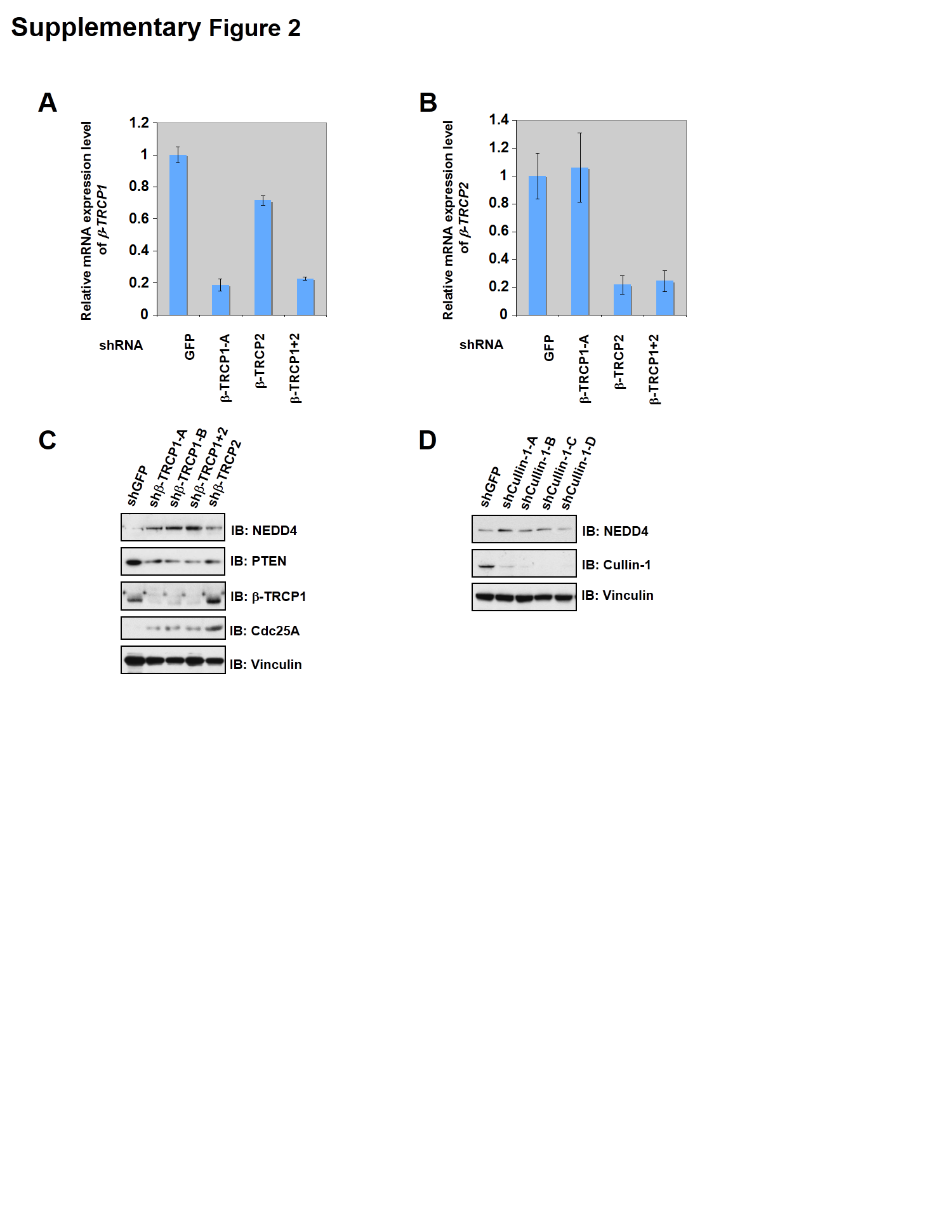


**Supplementary Figure 2**. **Depletion of β-TRCP or Cullin 1 increased NEDD4 protein levels.**

**(A-B)** Relative mRNA levels of β-TRCP1 (**A**) and β-TRCP2 (**B**) after the indicated shRNA treatment in HeLa cells were monitored by real-time RT-PCR analysis.

1. Immunoblot (IB) analysis of whole cell lysates (WCL) derived from 293T cells infected with shRNA constructs specific for GFP, β-TRCP1, β-TRCP2 or β-TRCP1+2, followed by selection with 1 μg/ml puromycin for three days to eliminate the non-infected cells.
2. IB analysis of WCL from PC3 cells transfected with shRNA specific for GFP or Cullin 1 followed by selection with 1 μg/ml puromycin for three days to eliminate the non-infected cells.


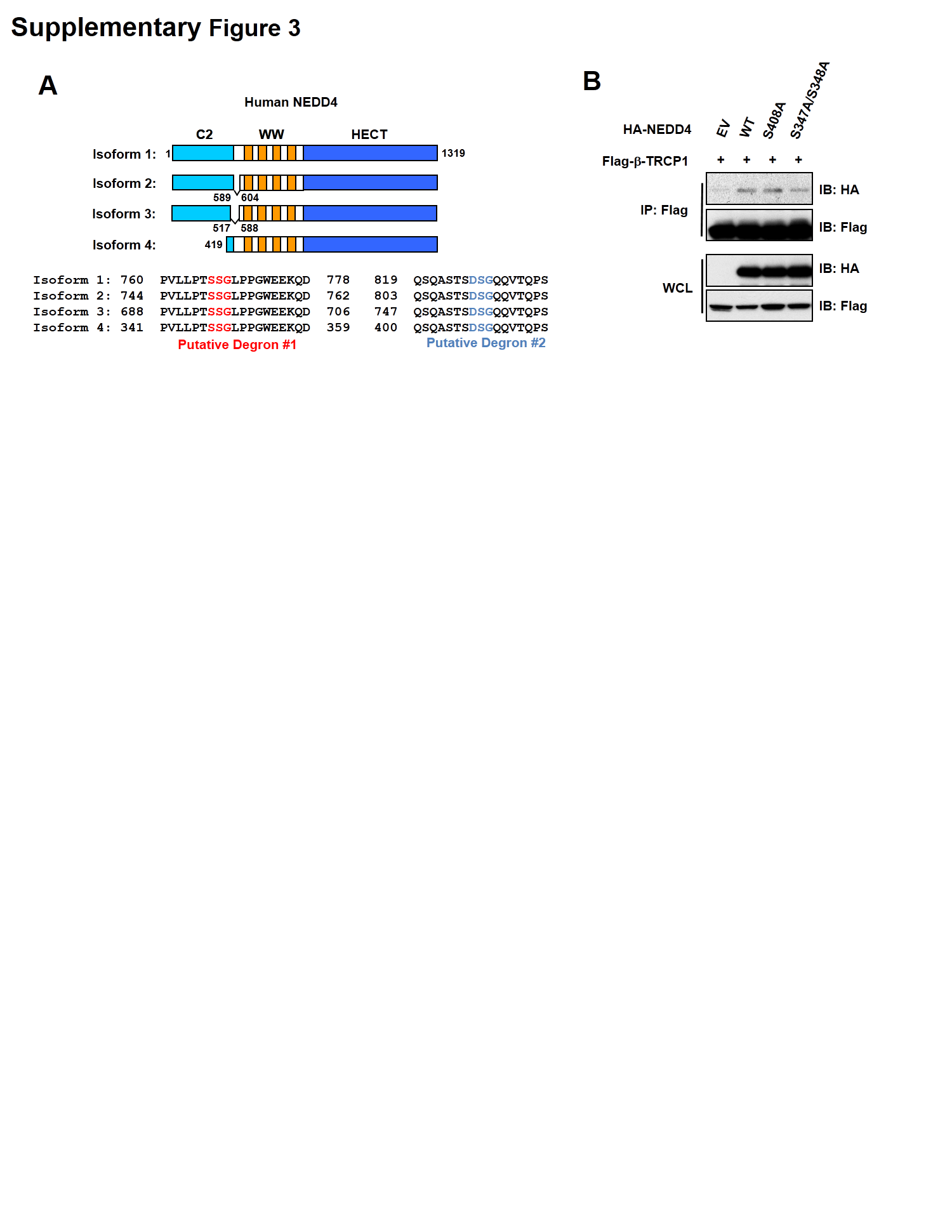


**Supplementary Figure 3**. **CKIδ phosphorylated NEDD4 at both S347 and S348 sites within the putative phospho-degron #1 to trigger its ubiquitination by SCF^β-TRCP^**.

**(A)** Alignment of the four human NEDD4 isoforms to illustrate that both putative degron #1 and putative degron #2 are present in all isoforms.

**(B)** Immunoblot analysis (IB) of whole cell lysates (WCL) and immunoprecipitates (IP) derived from 293T cells transfected with Flag–β-TRCP1 together with HA-WT–NEDD4, HA-S408A-NEDD4 or HA-S347A/S348A-NEDD4 as indicated.


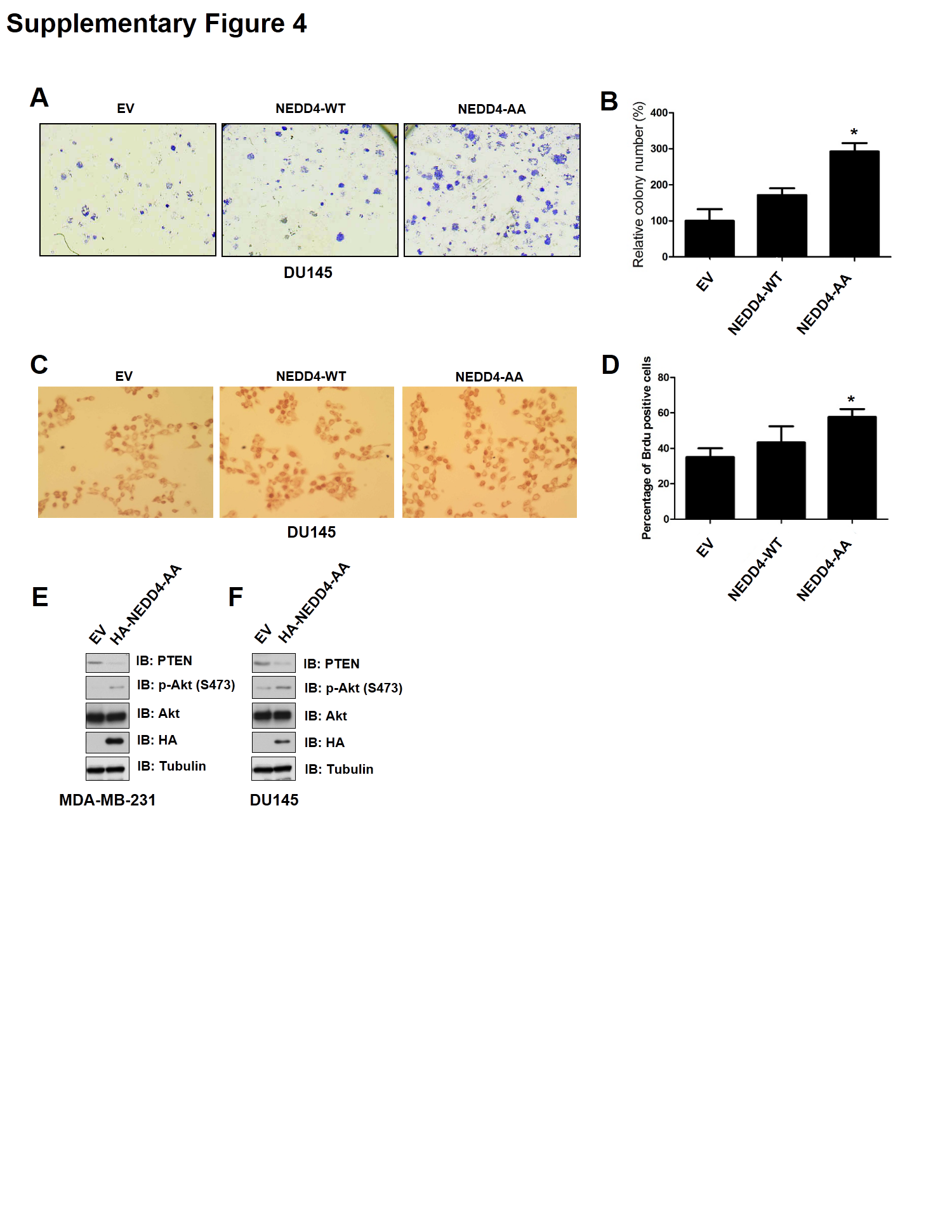


**Supplementary Figure 4**. **SCF^β-TRCP^-mediated degradation of NEDD4 modulated cell growth and migration.**

**(A-B)** Colony formation assay was performed in DU145 cells that were infected with EV, HA-wild-type (WT)-NEDD4 or HA-S347A/S348A (AA)-NEDD4 retroviral vectors followed by 3 days of puromycin (1 μg/ml) selection to eliminate the non-infected cells. After 4 days, the colonies were stained on the plates with crystal violet and counted (**A**). The numbers of surviving colonies were calculated as the average of triplicates (**B**). The error bars represented mean ± SD (*n* = 3), * *p*<0.05 (Student’s *t*-test), compared with cells expressing WT-NEDD4.

**(C-D)** BrdU labeling analysis was performed in DU145 cells that were infected with EV, HA-WT-NEDD4 or HA-AA-NEDD4 retroviral vectors followed by 3 days of puromycin (1 μg/ml) selection to eliminate the non-infected cells. Cells were incubated with BrdU and uridine for 48 hours and representative photographs of the stained cells were shown in (**C**). Percentage of BrdU positive cells was illustrated (**D**). The error bars represented mean ± SD (*n* = 3), * *p*<0.05 (Student’s *t*-test), compared with cells expressing WT-NEDD4.

**(E-F)** Immunoblot (IB) analysis of whole cell lysates (WCL) derived from MDA-MB-231 (**E**) and DU145 (**F**) cells infected with retroviral constructs encoding EV or HA-NEDD4-AA, followed by selection with 1 μg/ml puromycin for three days to eliminate the non-infected cells.
